# Selective Degradation of Target Proteins by Chimeric Small-Molecular Drugs, PROTACs and SNIPERs

**DOI:** 10.3390/ph13040074

**Published:** 2020-04-21

**Authors:** Minoru Ishikawa, Shusuke Tomoshige, Yosuke Demizu, Mikihiko Naito

**Affiliations:** 1 Graduate School of Life Sciences, Tohoku University, 2-1-1 Katahira, Aoba-ku, Sendai 980-8577, Japan; stomoshi@tohoku.ac.jp; 2 Division of Organic Chemistry, National Institute of Health Sciences, 3-25-26, Tonomachi, Kawasaki, Kanagawa 210-9501, Japan; demizu@nihs.go.jp; 3 Graduate School of Medical Life Science, Yokohama City University, 1-7-29, Yokohama, Kanagawa 230-0045, Japan; 4 Division of Molecular Target and Gene Therapy Products, National Institute of Health Sciences, 3-25-26, Tonomachi, Kawasaki, Kanagawa 210-9501, Japan; miki-naito@nihs.go.jp

**Keywords:** PROTACs, SNIPERs, chemical protein degradation

## Abstract

New therapeutic modalities are needed to address the problem of pathological but undruggable proteins. One possible approach is the induction of protein degradation by chimeric drugs composed of a ubiquitin ligase (E3) ligand coupled to a ligand for the target protein. This article reviews chimeric drugs that decrease the level of specific proteins such as proteolysis targeting chimeric molecules (PROTACs) and specific and nongenetic inhibitor of apoptosis protein (IAP)-dependent protein erasers (SNIPERs), which target proteins for proteasome-mediated degradation. We cover strategies for increasing the degradation activity induced by small molecules, and their scope for application to undruggable proteins.

## 1. Introduction

Conventional small-molecular drug discovery has relied on the lock-and-key theory, that is, discovery/optimization of small-molecular drugs that bind to target proteins and modulate their functions. For enzymes and receptors, there are many successful examples based on this approach, and various inhibitors, agonists, and antagonists have entered clinical use. However, it is difficult to modulate the functions of other proteins, such as substrate-binding proteins, aggregation-prone proteins, protein–protein complexes, and so on, because few drug-like modulators of such proteins have been identified so far. An estimated 60% of small-molecular drug discovery projects fail during hit-to-lead development because the biological target protein is found to be “undruggable”, that is, the protein cannot bind to compounds with appropriate drug-like properties [1]. Indeed, a genomic analysis has concluded that only 10% of genes in the human genome are druggable, and only 5% are both druggable and relevant to disease [2]. Therefore, there has been increasing interest in the development of new therapeutic modalities in recent decades. 

In addition to potential pharmaceutical applications, techniques for decreasing the expression levels of proteins are useful for analysis of the functions of proteins of interest. For example, RNAi [3], CRISPR/cas9 [4], and gene knockout have been widely used in the fields of biochemistry, molecular biology, and so on. Nevertheless, chemical techniques to decrease target proteins after translation would have several advantages, such as: (i) better pharmacokinetics, especially absorption and metabolic stability, resulting in easy administration of compounds, in contrast to genetic techniques, (ii) capability of post-translationally regulating the protein levels; i.e., the protein levels are reduced independently of the protein half life, in general. This review article covers chimeric drugs that decrease the levels of target proteins via inhibitor of apoptosis proteins (IAP)-mediated ubiquitination, focusing in particular upon our work on protein degraders termed specific and non-genetic inhibitor of apoptosis protein (IAP)-dependent protein erasers (SNIPERs) in addition to important proteolysis targeting chimeric molecules (PROTACs). 

## 2. Physiological Degradation of Proteins via the Ubiquitin-Proteasome System

Physiological degradation of proteins via the ubiquitin-proteasome system is crucial for regulating cellular functions, including the cell cycle, immune responses, and signal transduction [5,6]. In general, protein ubiquitination is mediated by sequential reactions of a ubiquitin-activating enzyme (E1), a ubiquitin-conjugating enzyme (E2), and a ubiquitin ligase (E3) [5]. First, the E1 enzyme (there are two in the human genome) covalently attaches to ubiquitin [7] via a thioester bond in an ATP-dependent manner [8]. Next, E1 transfers ubiquitin onto an E2 enzyme (there are about 40 in the human genome) [9]. Finally, E2 binds a substrate-bound E3 ligase (there are about 600 in the human genome [10]) to transfer ubiquitin onto a lysine residue of the substrate [9]. Subsequent repeated ubiquitination at lysine 48 on ubiquitin creates a K48-linked polyubiquitin chain [11], and the K48-polyubiquitinated proteins are recognized and degraded by proteasome [12,13]. Many E3 ligases have been reported, and it is thought that different E3 ligases have different specificities, that is, they distinguish various proteins that are to be ubiquitinated. In addition to the K48 polyubiquitin chain, ubiquitin can be conjugated through their lysine residues (K6, K11, K27, K29, K33, and K63) or the N-terminal methionine residue (M1), and different types ubiquitination often elicit distinct functions [14]. K63 chain is involved in processes such as endocytic trafficking, inflammation [15] and DNA repair [16], and M1 chain is involved in an activation of NF-kB [17].

## 3. Peptidic PROTACs

Pioneering research providing a proof of principle regarding induction of protein degradation was reported in 2001 [18]. In that work, the chimeric drug **1** composed of a peptide sequence that is recognized by a ubiquitin ligase (SCF^βTRCP^) and a ligand for a target protein (methionine aminopeptidase: MetAP) formed an artificial complex between SCF and MetAP, and induced degradation of MetAP in *Xenopus* extracts (Table 1). This peptidic chimeric molecule **1** was termed a PROTAC (proteolysis targeting chimeric molecule). In 2004, another peptidic PROTAC **2** consisting of a peptide sequence that is recognized by a ubiquitin ligase (von Hippel-Lindau: VHL), a ligand for a target protein (FK506 binding protein: FKBP), and a poly-D-arginine tag as a cell-penetrating peptide was reported [19]. This PROTAC induced degradation of FKBP in living cells. However, the membrane permeability or stability of peptide structures is often inadequate for biological studies and therapeutic applications. In this context, a non-peptide chimeric drug **3** composed of a ligand (nutlin-3) for E3 ligase (murine double minute 2: MDM2) and a ligand for androgen receptor (AR) was shown to decrease AR levels in 2008 [20]. This hybrid small molecule **3** has been referred to as the first small-molecular PROTAC in review articles. However, this molecule **3** is recognized as a degrader of the endogenous protein substrate because AR is an endogenous protein substrate of MDM2 [21]. Induction of an artificial protein complex must be more challenging than induction of an endogenous protein complex. Moreover, MDM2-bound nutlin-3 naturally enhances ubiquitination of AR [22]. Therefore, additional data to compare the AR levels after nutlin-3 treatment and chimeric drug **3** treatment would be needed to determine whether the decrease of AR is associated with the nutlin-3 derivative or the PROTAC [23,24]. 

## 4. IAP-Mediated Small-Molecular Protein Degraders

Based on our above analysis of the pioneering research on PROTACs, we set out to develop a practical chemical protein knockdown, that is, induction of an artificial (non-physiological) complex between E3 and a target protein, followed by degradation of the target protein in response to small molecules. For this purpose, we focused on cellular inhibitor of apoptosis protein 1 (cIAP1), which is one of the IAPs [47]. It is ubiquitously expressed, but is overexpressed in certain tumor cells. cIAP1 contains three baculoviral IAP repeat (BIR) domains that interact with its substrates, and one really interesting new gene (RING) finger domain that is involved in ubiquitin ligase activity. Thus, cIAP1 is classified as a RING-finger E3 ligase [48,49]. cIAP1 promotes ubiquitination and proteasomal degradation of its substrate proteins, including receptor-interacting protein 1 (RIP1), NF-κB-inducing kinase (NIK), and cIAP1 itself [50,51]. Upon ubiquitination of RIP1 and NIK, cIAP1 inhibits apoptosis induced by a variety of stimuli via modulation of canonical and non-canonical NF-kB pathways [52]. In addition, overexpression of cIAP1 correlates with resistance to radiotherapy and chemotherapy in various cancers [53]. 

**Figure 1:**
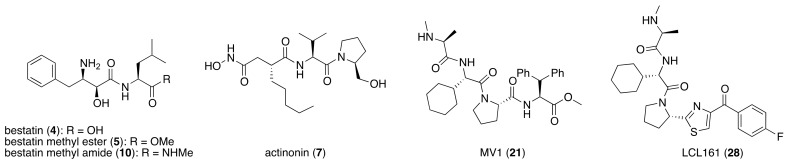
Chemical structures of small-molecular cIAP1 ligands.

Bestatin (**4**, Figure 1), *N*-[(2*S*,3*R*)-3-amino-2-hydroxy-4-phenylbutanoyl]-l-leucine, isolated from *Streptomyces olivoreticuli* in 1976 [54], is a competitive inhibitor of aminopeptidases, including aminopeptidase B and aminopeptidase N, and is clinically used to treat acute myeloid leukemia and lymphedema [55]. In 2008, bestatin methyl ester (**5**) was found to bind to the BIR3 domain of cIAP1 and to promote ubiquitination and degradation of cIAP1 [56]. Importantly, the structure–activity relationships of bestatin analogs for cIAP1 degradation and amino peptidase inhibition are different. For example, bestatin (**4**) is a more potent inhibitor of aminopeptidase than bestatin methyl ester (**5**) (Table 2), whereas 15 μM bestatin methyl ester (**5**) induces cIAP1 degradation more efficiently than 15 μM bestatin (**4**) [56]. Introduction of a para-hydroxy group into bestatin (**6**) yields a more potent aminopeptidase inhibitor than bestatin (**4**), whereas 15 μM bestatin (**4**) induces cIAP1 degradation more efficiently than 15 μM hydroxybestatin (**6**). Chemical evidence of association of bestatin (**4**) and the BIR3 domain in cIAP1 was obtained by means of fluorescence polarization assay and photoaffinity labeling assay [57]. As another cIAP1 ligand, actinonin (**7**, Figure 1) [58], an antibiotic and an inhibitor of leucine aminopeptidase and aminopeptidase N, was found to possess similar cIAP1 degradation-promoting activity. Structure–activity relationship studies led to a hybrid compound of bestatin (**4**) and actinonin (**7**) that promotes degradation of cIAP1 with the IC_50_ value of 0.5 μM [59]. 

Over 600 kinds of E3 ligases catalyze substrate-specific ubiquitination [10]. However, the ubiquitination of cIAP1 occurs via formation of its homodimer [60]. This report led our initial idea that an artificial (non-physiological) protein complex between cIAP1 and an intercellular target protein induce cIAP1-mediated ubiquitination and proteasomal degradation of the target protein under physiological conditions. To form the artificial protein complex, the twin-drug strategy [61] was selected, and a hybrid molecule consisting of bestatin methyl ester (**5**) coupled to a ligand for a target protein was designed.

To set a target disease, possible advantages of the chemical protein knockdown approach compared with the conventional lock-and-key strategy were discussed. They are (i) synergistic/additive effect of inhibition and decrease of the target protein when the inhibitor is conjugated, (ii) usage of a ligand that does not modulate the functions of the target proteins, (iii) synergistic/additive effect by modulation of the function of the ubiquitin ligase, (iv) longer duration of action than a conventional reversible inhibitor, (v) a catalytic effect that might enable the use of a low concentration of drug or a ligand with weak affinity. To suppress the appearance of drug resistance, a combination of inhibition and target protein decrease might be more effective. As bestatin methyl ester (**5**) induces a decrease in the level of cancer-related protein cIAP1, chimeric drugs based on bestatin esters might show an additive effect in cancer treatment. Thus, for proof of concept, cancer was focused as a target. Specifically, cellular retinoic acid binding protein (CRABP) was selected as the first target protein because (i) CRABP-II induces migration of neuroblastoma cells independently of its endogenous ligand, all-*trans* retinoic acid (ATRA, **8**) [62]; (ii) no direct CRABP inhibitor has been reported; (iii) CRABPs bind strongly to ATRA (the *K*_d_ values of CRABP-I and CRABP-II are 5 and 60 nM, respectively [63]); and (iv) sufficient information is available about the effect of linker position on the binding affinity, as discussed below.

ATRA (**8**) also binds to retinoic acid receptor (RAR), and is clinically utilized for the treatment of acute promyelocytic leukemia [64]. To improve the degradation selectivity for CRABP over RAR, the linker was introduced at the allylic position of the cyclohexenyl group of ATRA because (i) the cyclohexenyl group is located outside the binding pocket of CRABP [65], but inside of that of RAR [66], and (ii) introduction of the linker at that position retained the binding affinity toward CRABP, but not towards RAR [67]. In addition, as mentioned above, bulky substituents at the ester moiety of bestatin methyl ester (**5**) do not markedly affect the binding affinity to cIAP1. Thus, a linker including triethylene glycol was used to link the ester position of bestatin methyl ester (**5**) to the allylic position of ATRA (**8**), affording compound **9** (Table 1). Since the linker length is likely to influence the ubiquitination efficiency of target proteins, two other analogs possessing a shorter diethylene glycol linker and a longer tetraethylene glycol linker were also synthesized. Compound **9** decreased CRABP-I and CRABP-II levels in living cells. The linker length affected the selectivity. The compound (1 μM) possessing the shorter linker decreased CRABP-I, whereas the compound (0.1 μM) possessing the longer linker decreased CRABP-II. Compound **9** decreased cIAP1 similarly to bestatin methyl ester (**5**). In mechanistic analysis, co-treatment with excess bestatin methyl ester (**5**), as well as a cIAP1 RNAi experiment [68], revealed that the decrease of CRABP was cIAP1-mediated. In addition, formation of a complex between cIAP1 and CRABP was detected by GST pull down assay, ubiquitinated CRABP was detected by means of immunoprecipitation [68], and proteasomal degradation was confirmed by co-treatment with a proteasome inhibitor. These results support our hypothesis that the hybrid molecule **9** forms an artificial ternary complex with cIAP1 and CRABP, in which cIAP1 ubiquitinates CRABP, leading to its degradation by proteasome. We confirmed that **9** did not induce degradation of RAR and did not bind to RARs, as expected. Compound **9** inhibited migration of neuroblastoma cells in a dose-dependent manner, indicating that chemical protein knockdown in cells can induce phenotypic change. Thus, targeted cancer therapy via decrease of cancer-related protein might be feasible, even if no inhibitor has been identified. The first small molecules that degrade target proteins in living cells were reported in 2010 [25]. Here, it should be emphasized that CRABP-II can be regarded as an undruggable protein, because all-*trans* retinoic acid is classified as a functionally neutral ligand for CRABP, and the chemical knockdown of an undruggable protein with the use of a functionally neutral ligand clearly opens up a novel modality. Following the success of induction of E3-mediated selective degradation of target proteins by small molecules (protein knockdown), small molecules that induce protein knockdown were designated as SNIPERs.

## 5. Selective Knockdown of Target Protein

As the hybrid compound **9** degrades both cIAP1 and CRABP, selective target protein degradation was next investigated. Bestatin methylamide (**10**) was found to bind to cIAP1, but not decrease it [56,60]. Based on this finding, a conjugated molecule **11** consisting of **10** and ATRA (**8**) was designed as a selective degrader of CRABP over cIAP1. Indeed, the synthesized amide **11** decreased CRABP-II at 0.1 μM, but not cIAP1 even at 10 μM. In addition, the functions of cIAP1 (activation of caspases and apoptosis) were affected by treatment with bestatin methyl ester (**5**) and **9**, but not amide **11**. CRABP-II increases expression of the oncogene MycN that inhibits apoptosis of neuroblastoma cells [62]. Amide **11** decreased MycN levels concomitantly with a decrease of CRABP-II in neuroblastoma IMR-32 cells, whereas **11** hardly inhibited proliferation of HT1080 and MCF-7 cells, which express CRABP-II and cIAP1, but not MycN. These results suggest that the inhibition of cell proliferation by amide **11** is specific for MycN-expressing cells. In addition, double knockdown of cIAP1 and CRABP-II (single treatment with ester **9**, as well as the combination of amide **11** and bestatin methyl ester (**5**)) showed a stronger anti-proliferative effect on neuroblastoma cells than single knockdown of CRABP-II and cIAP1. These results reported in 2011 [26] suggest that double knockdown of both cIAP1 and a cancer-related protein (in this case CRABP-II) is more effective for cancer treatment than knockdown of the cancer-related protein alone.

## 6. Scope of Protein Knockdown

The bestatin-conjugated small-molecular protein knockdown strategy is applicable to receptors. Estrogen receptor (ER), androgen receptor (AR), and RAR are members of the nuclear receptor superfamily of ligand-dependent transcription factors, and estrone and dihydrotestosterone are endogenous agonists for ER and AR, respectively. Treatment of living cells with 30 μM endogenous agonist-conjugated **12** and **13** decreased the ER and AR levels, respectively [27]. Non-steroidal ligands for nuclear receptors are also applicable for chemical protein knockdown. The non-steroidal RAR agonist Ch55, which hardly binds to CRABP-II, was selected with the aim of achieving selective degradation of RAR over CRABP. The linker position of Ch55 was determined on the basis of previously determined structure–activity relationships. Treatment of living cells with 10 μM **14** decreased the RAR level, but not the CRABP-II level [27]. As for ER, hydroxytamoxifen is a non-steroidal ER antagonist, and an active metabolite of tamoxifen, which is used to treat breast cancer. Treatment with 10 μM non-steroidal hydroxytamoxifen-conjugated **15** decreased the ER level [69]. A mechanistic analysis revealed that **15** induces the production of reactive oxygen species (ROS) and causes necrotic cell death of ER-expressing breast cancer cells [29]. As for AR, hybrid molecules composed of five kinds of non-steroidal antagonists and bestatin methylamide (**10**) were reported [45]. These compounds, including **16**, bound to androgen receptor with IC_50_ values of 0.027, 0.19, 0.19, 1.1, and >10 μM, and decreased AR with the DC_50_ values of 21.6, 11.7, 20.5, >30, >30 μM, respectively. These results might suggest that incorporation of a ligand with higher binding affinity for the target protein is preferable to more potent protein knockdown activity.

As a further scope of protein knockdown, subcellular localization, and application to enzymes were investigated. The subcellular localization of CRABP-II is mainly the cytosol, suggesting that SNIPERs can target cytosolic proteins. Next, to check the scope of subcellular localization, HaloTag system was utilized. HaloTag is an artificial dehalogenase enzyme that recognizes and binds covalently to 1-chloroalkane, and is widely utilized as a protein tag in chemical biology [70]. The hybrid small molecule **17** composed of bestatin methylamide (**10**) and chlorohexane decreased the levels of two artificial enzymes, HaloTag-fused cAMP responsive element binding protein 1 (HaloTag-CREB1) and HaloTag-fused c-jun (HaloTag-c-jun) [30]. These fused proteins are localized mostly in the nucleus, suggesting that the chemical protein knockdown approach could be applicable to nuclear proteins. To check the scope of subcellular localization, cells expressing CRABP-II in cytosol, nucleus, plasma membrane, and mitochondria were generated by inserting appropriate localization signal sequences [71]. The degrader **9** induced degradation of cytosolic, nuclear, membrane-localized, and mitochondrial CRABP-II in the cells. However, it was also suggested that nuclear and mitochondrial CRABP-II is decreased by cIAP1-independent but proteasome-dependent degradation [71]. In 2016, the hybrid molecule **18** consisting of bestatin methylamide (**10**) and an analog of the BCR-ABL inhibitor imatinib was shown to decrease BCR-ABL [37] suggesting that chemical protein knockdown is applicable to native enzymes.

Neurodegenerative diseases, including Alzheimer’s disease, Parkinson’s disease, and polyglutamine (polyQ) diseases, exhibit a variety of symptoms and involve various disease-related proteins, such as amyloid β, α-synuclein, and polyQ. However, these diseases appear to have a common pathogenic mechanism, that is, disease-related proteins are misfolded into β-sheet-rich monomers that are prone to aggregate in neuronal cells. The soluble oligomer aggregates cause neuronal cell death, leading to a variety of symptoms. For example, Huntington’s disease (HD) is an intractable autosomal dominant disorder caused by aggregation-prone mutant huntingtin (mHtt), which has an extended N-terminal polyQ tract. Therefore, HD is a good candidate for testing novel approaches to inhibit oligomer aggregates. But, there are two difficulties in applying chemical knockdown to aggregation-prone proteins. First, there are many reports regarding dysfunction of the ubiquitin-proteasome system in neurodegenerative diseases, but it has also been suggested that expression of E3 ameliorates symptoms, and a review points out that the situation remains unclear [72]. The second issue is that few small-molecular ligands for disease-related proteins have been identified so far. Instead, we designed hybrid molecules consisting of bestatin methylamide (**10**) and a probe for aggregates as degraders of aggregation-prone proteins. The probes BTA and PDB were selected because (i) they do not contain ionic structure, (ii) they stain aggregates in vivo, and (iii) sufficient information is available about the effects of linker position. In 2017, it was reported that the synthetically designed drugs **19** and **20** decreased mHtt in fibroblasts derived from HD patients, as well as in Hela cells transfected with mHtt exon-1 with a much longer polyQ repeat 145 [40]. Mechanistic analysis revealed that (i) **20** did not decrease *HTT* mRNA, (ii) an artificial complex between cIAP1 and aggregates was detected by means of ELISA, (iii) a negative control compound (an analog in which the hydroxy group and amino group on bestatin were replaced with hydrogen atoms, and which does not bind to cIAP1) did not reduce mHtt level, and iv) proteasomal degradation of mHtt was confirmed by co-treatment with a proteasome inhibitor MG132. Furthermore, **20** decreased the amount of mHtt aggregates in cells. Disease-related proteins involved in other polyglutamine diseases, including atrophin-1, ataxin-7 and ataxin-3, were also decreased by compounds **19** and **20** [46], suggesting that hybrid compounds composed of an E3 ligand and a probe for aggregates might be generally effective for neurodegenerative disorders caused by aggregation-prone proteins. This universal effectiveness might achieve not only general treatment for these incurable diseases, but also better efficacy to diseases caused by multiple aggregation-prone proteins, such as Alzheimer’s disease.

## 7. IAPs Pan Antagonist-Mediated Protein Knockdown

Among IAPs, not only cIAP1, but also cIAP2 and X-linked inhibitor of apoptosis protein (XIAP) are overexpressed in certain tumor cells and inhibit apoptosis induced by a variety of stimuli [47]. XIAP, cIAP1, and cIAP2 show ubiquitin ligase (E3) activity and they promote proteasomal degradation of their substrate proteins, including caspases, which are effectors of apoptosis. Therefore, inhibition of these IAPs is a potential strategy for cancer treatment. The mitochondrial protein Smac (second mitochondria-derived activator of caspases) is an endogenous IAP antagonist that binds to the BIR domains of IAPs through the Ala-Val-Pro-Ile (AVPI) tetrapeptide sequence on Smac. Potent and cell-permeable small-molecular IAP antagonists mimicking AVPI peptide are in clinical trials for cancer treatment [73]. In addition, it was also reported that pan antagonism of both cIAPs and XIAP induces cancer cell death more efficiently than antagonists of cIAPs alone [74].

Since double knockdown of both cIAP1 and a cancer-related protein was an effective approach, a chimeric drug incorporating an IAPs pan antagonist seems likely to be a promising approach for cancer therapy. In addition, this design concept is expected that simultaneous utilization of multiple ubiquitin ligases results in more efficient degradation of the target protein. Indeed, the CARBP-II ligand all-*trans* retinoic acid (**8**) and IAPs pan antagonist MV1 (**21**), which induces cancer cell death, were linked to afford the cell-permeable hybrid molecule **22**, which was found to decrease CARBP-II at a lower concentration and showed greater anti-proliferative activity against neuroblastoma IMR-32 cells, as compared with bestatin-conjugate **9** in 2012 [28].

A hybrid molecule **23** consisting of IAPs pan antagonist MV1 (**21**), a cognate of the LXXLL (L: leucine, X: any amino acid) peptide fragment on coactivator protein that binds to ER, and a hepta-arginine tag as a cell-penetrating peptide was reported in 2016 [35]. It is noteworthy that this peptide fragment binds to a different site from the conventional agonist/antagonist binding pocket. The peptide decreased ER in cells, suggesting that a chemical knockdown approach might be effective for not only proteins amenable to the lock-and-key approach, but also undruggable proteins. In other words, a ligand that binds to any site, and a ligand that either modulates or does not modulate the function(s) of the protein can be utilized for chemical protein knockdown. Another example of degradation of an undruggable protein by a peptide-based hybrid molecule was reported in 2017 [38]. A hybrid molecule **24** consisting of IAPs pan antagonist MV1 (**21**) and a stapled peptide that binds to transcriptional factor NOTCH1 induced a decrease in NOTCH1.

As miscellaneous examples of SNIPERs, hybrid small molecules, including **25** composed of IAPs pan antagonist MV1 (**21**) and chlorohexane, decreased HaloTag-fused proteins in living cells more potently than did bestatin-conjugate **17 [36]**. His-Tag-fused proteins were decreased by a combination of carrier peptide (His-Tag-fused cell-penetrating peptide) and a hybrid compound **26** composed of IAPs pan antagonist MV1 (**21**), nickel nitrilotriacetic acid (Ni-NTA) as a His-tag ligand, and a maleimide moiety to form a covalent bond to a Cys residue in a His-tagged target protein [44]. In this study, all the components were indispensable; in the absence of the maleimide moiety or the carrier peptide, no decrease of His-Tag fused protein was observed. Next, a hybrid compound **27** composed of MV1 (**21**) and the aggregates probe PDB successfully decreased mHtt levels in living cells [42]. However, although the affinity of MV1 (**21**) for cIAP1 is greater than that of bestatin methylamide (**10**), the efficacy of mHtt degradation by the MV1-conjugate **27** was less than that of bestatin-conjugate **19**, supporting the idea that the linker length between the ligand and probe might be an important determinant of efficacy, as mentioned above.

In vivo chemical protein knockdown utilizing IAP was first achieved in 2017 with the use of another IAPs pan antagonist LCL161 (**28**) [39]. A hybrid compound **29** composed of LCL161 (**28**) and ER antagonist 4-hydroxytamoxifen decreased the ER level in cells at a 10-times-lower concentration compared with the MV1-linked compound. The knockdown activity was evaluated in vivo, using female BALB/c mice intraperitoneally injected with **29** (10 or 30 mg/kg), and a decrease of ER level in the ovaries was observed. A similar effect was seen in nude mice bearing MCF-7 breast tumor xenografts. The Cmax and AUC_0-24h_ of **29** (after intraperitoneal injection at 10 mg/kg) were 1970 ng/mL and 16923 ng h/mL, respectively. When nude mice with MCF-7 breast tumor xenografts were treated with **29** (intraperitoneal injection at 30 mg/kg daily for 14 days), the tumor volume was reduced. Notably, there was no obvious toxicity, including body weight change, throughout the 2 weeks of administration of **29**.

To investigate the generality of LCL161-conjugated molecules, LCL161 (**28**) was conjugated with dasatinib, JQ-1 and phosphodiesterase 4 (PDE4) inhibitor, ligands of BCR-ABL, bromodomain containing protein 4 (BRD4), and PDE4, respectively [39]. These LCL161-conjugated chimeric drugs **30**-**32** generally showed efficient knockdown of their target proteins at nanomolar concentrations. In all cases, a hook effect [75], that is, reduced degradation at high concentrations of degrader, was observed as well. The reason for the hook effect is that when the concentration of the degrader is high, formation of the ternary complex of E3-degrader-target protein is inhibited because of the high concentrations of binary complexes (degrader-E3 and degrader-target protein). A hybrid molecule **33** consisting of LCL161 (**28**) and a non-steroidal AR antagonist also decreased the AR level in cells, but at a higher concentration (3 μM) [45].

Structure–degradation activity relationships of ER degrader **29** were investigated by substitution of the E3 ligand moiety with various reported IAPs pan antagonists [43]. Among them, several hybrid molecules decreased ER, cIAP1, and XIAP. Although **29** preferentially recruits XIAP to degrade ER, the degradation activity of ER was independent of the binding affinity for XIAP. On the other hand, the degradation activity of cIAP1 seems to be roughly dependent on the binding affinity of the hybrid molecules for cIAP1. Interestingly, degradation of XIAP was induced by the hybrid molecules, but not by IAPs pan antagonists alone or by a mixture of IAPs pan antagonist and 4-hydroxytamoxifen, suggesting that the ternary complex (E3-degrader-ER) is required for XIAP degradation. Consistent with this hypothesis, XIAP degradation by **29** was not observed in ER-negative cells. In an in vivo MCF-7 tumor xenograft model, **34** showed superior antitumor activity to **29**.

Both the degradation and inhibition of BCR-ABL inhibit the proliferation of cancer cells, but the effects of degradation were sustained when the drugs were removed following short-term treatment [76]. Chronic myelogenous leukemia (CML) cells treated with BCR-ABL inhibitors resumed proliferation immediately after drug removal, whereas cells treated with **32** could not proliferate and eventually underwent cell death under the same conditions. This result suggests that degraders may induce longer-lasting effects than conventional inhibitors.

Resistance to kinase inhibitors is often attributed to mutations in the kinase domain, so this approach could be used as an alternative strategy to overcome drug resistance, by capturing different domains of oncogenic kinases to induce their degradation. In detail, small-molecular degrader **35** that binds to the allosteric site of BCR-ABL also degrades BCR-ABL, and inhibits proliferation of BCR-ABL-expressing cells [41].

## 8. Cereblon and VHL as Components of Small-Molecular PROTACs

In 2015, several small-molecular chimeric drugs **36**-**40** composed of E3 ligands other than IAPs and ligands for target proteins BRD4, receptor-interacting serine/ threonine kinase (RIPK) and estrogen-related receptor (ERR) were reported. In these studies, two types of E3 ligands were employed, cereblon ligand thalidomide [31,33] and VHL ligand [32,34]. Among them, PROTACs **38** and **39** were the first to achieve potent degradation of the target proteins in cells with DC_50_ values of nanomolar order. Further, **36** and **40** were the first to show efficacy in animal models in vivo after intraperitoneal treatment. Since then, research on small-molecular degraders has been increased rapidly. Recent progress in studies of small-molecular PROTACs has been reviewed elsewhere [77,78,79].

## 9. Conclusions

This review article summarizes work over the past decade on protein degraders that induce IAP-mediated ubiquitination. Chemical protein knockdown, that is, the induction of protein degradation by chimeric drugs composed of an E3 ligand and a target protein ligand, now represents a highly promising new modality for drug discovery. We reported the first small molecules that induce E3-mediated ubiquitination and degradation of a target protein. Both selective knockdown of the target protein, and double knockdown of the target protein/cIAP1 were achieved by changing the chemical structure of the cIAP1 ligand. Indeed, double protein knockdown of IAPs and cancer-related proteins is a promising approach for cancer treatment. This is a major advantage over other PROTACs that utilize the ubiquitin ligases VHL and cereblon. As regarding E3 ligase ligands, cIAP1 ligands (bestatin analogs) and IAPs pan antagonists have been utilized for chemical protein knockdown. Hybrid molecules consisting of bestatin required relatively high concentrations to achieve degradation, but an improvement of degradation efficacy was obtained with the use of IAPs pan antagonists. In particular, potent degradation with DC_50_ values of nanomolar order in cells, and efficacy in animal models after intraperitoneal treatment were achieved with IAPs pan antagonist LCL161-conjugated molecules. IAP-mediated protein degradation has been applied to enzymes, receptors, substrate-binding proteins, and aggregation-prone proteins. Notably, small-molecular degraders could degrade various undruggable proteins, including aggregation-prone proteins associated with neurodegenerative disorders, and proteins that bind only functionally neutral ligand. Proteins that bind peptidic ligands at other sites than the conventional ligand-binding sites have been degraded, and protein degradation with the use of an allosteric inhibitor has also been achieved. Specificity for subcellular location is also possible, as IAP-mediated protein degradation has been applied to proteins located in cytosol, nucleus, cell membrane, and mitochondria. Notably, IAP-mediated protein degraders show longer-lasting effects than conventional inhibitors. All these results indicate that chemical protein knockdown by chimeric drugs can reach beyond the boundaries imposed by conventional drug discovery based on the lock-and-key theory.

## Figures and Tables

**Table 1:** Timeline, chemical structures, and biological activities of IAP-mediated protein degraders along with other representative E3-mediated protein degraders.

Year	E3	Target Class	Target Protein	Molecular Weight	Compd No.	Chemical Structure	Efficacy in Cells	Efficacy In Vivo	Ref
2001	SCF^βTRCP^	enzyme	MetAP	1697	**1**	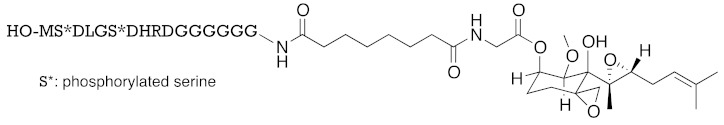	-	-	[18]
2004	VHL	Protein-protein interaction	FKBP	2894	**2**	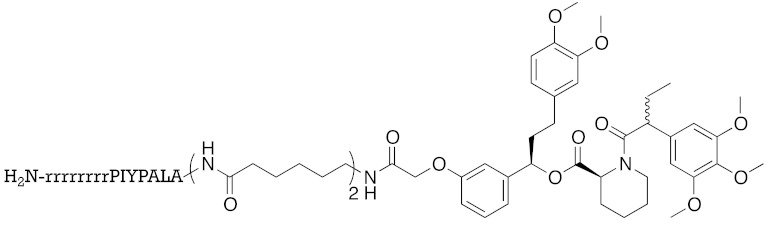	25 μM	-	[19]
2008	MDM2	Endogenous substrate of E3	AR	1210	**3**	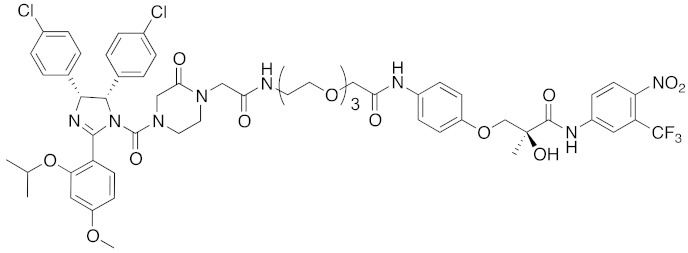	10 μM	-	[20]
2010	cIAP1	E3/ binding protein (functionally neutral ligand)	cIAP1/ CRABP-II	809	**9**	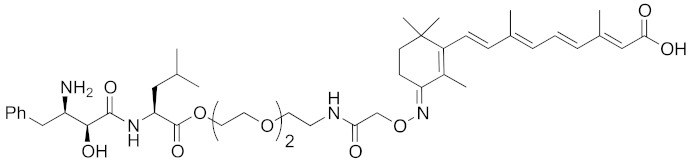	1 μM	-	[25]
2011	cIAP1	Binding protein	CRABP-II	808	**11**	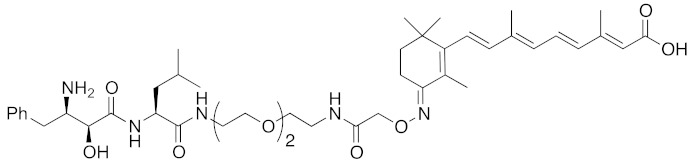	1 μM	-	[26]
2011	cIAP1	receptor	ER	764	**12**	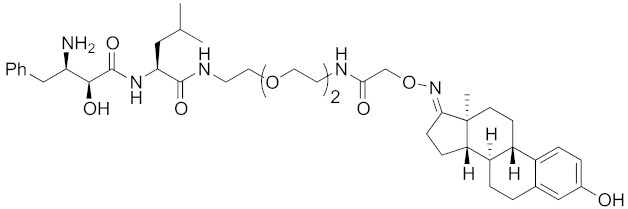	30 μM	-	[27]
2011	cIAP1	receptor	AR	770	**13**	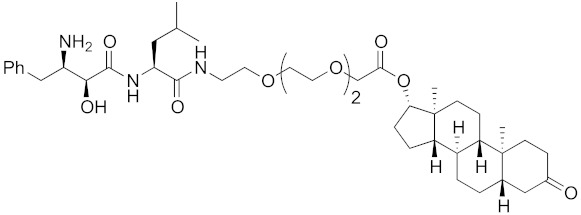	30 μM	-	[27]
2011	cIAP1	receptor	RAR	917	**14**	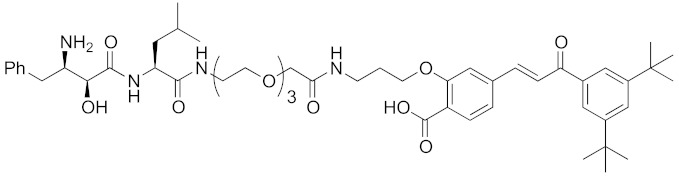	10 μM	-	[27]
2012	IAPs	E3/ binding protein	cIAP1/ CRABP-II	1062	**22**	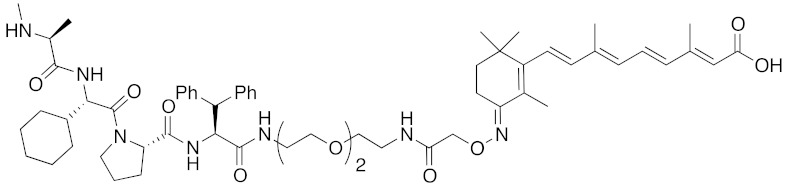	100 nM	-	[28]
2012	cIAP1	receptor	ER	763	**15**	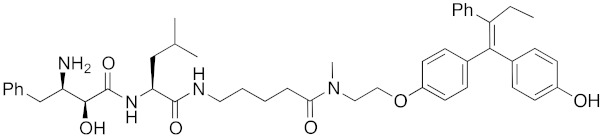	10 μM	-	[29]
2015	cIAP1	Enzyme/tag	HaloTag- fused proteins	602	**17**	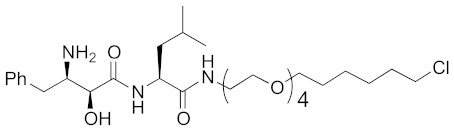	10 μM	-	[30]
2015	Celebron	others	BRD4	785	**36**	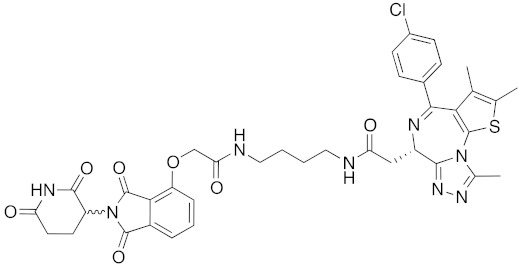	100 nM	50 mg/kg, IP	[31]
2015	VHL	others	BRD4	1002	**37**	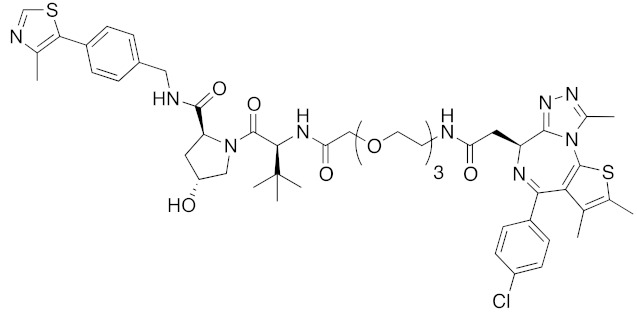	100 nM	-	[32]
2015	celebron	others	BRD4	923	**38**	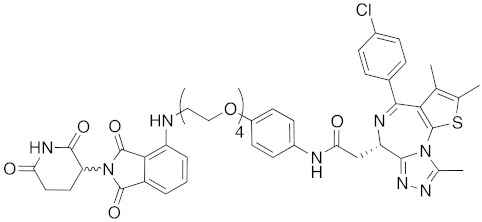	0.3 nM	-	[33]
2015	VHL	enzyme	RIPK	1060	**39**	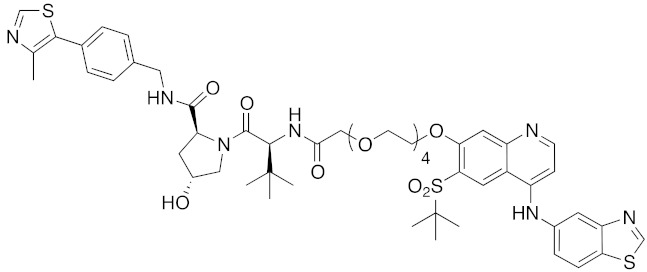	3 nM	-	[34]
2015	VHL	receptor	ERR	949	**40**	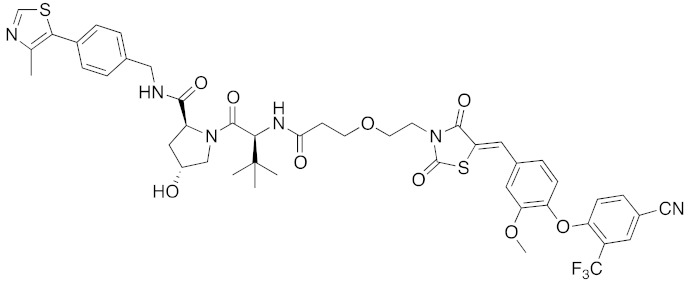	100 nM	100 mg/kg, ip	[34]
2016	IAPs	Receptor (coactivator binding site)	ER	3265	**23**	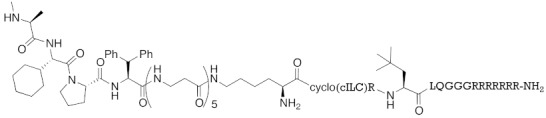	20 μM	-	[35]
2016	IAPs	Enzyme/tag	HaloTag- fused protein	834	**25**	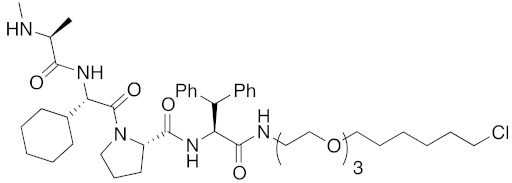	1 μM	-	[36]
2016	cIAP1	enzyme	BCR-ABL	828	**18**	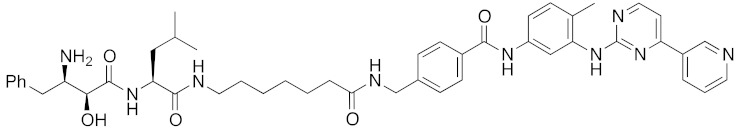	30 μM	-	[37]
2017	IAPs	enzyme (undruggable)	Notch1	2989	**24**	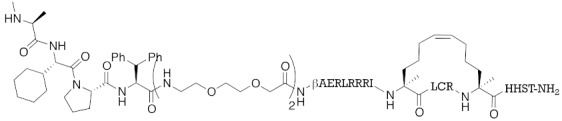	100 μM	-	[38]
2017	IAPs	E3/ receptor	ER	1044	**29**	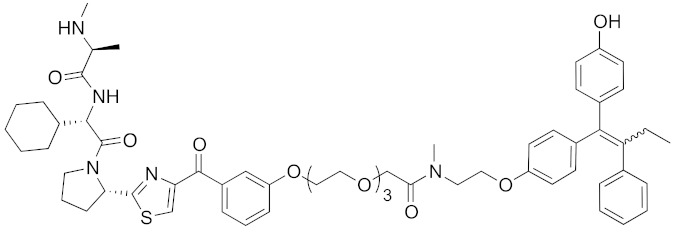	3 nM	10 mg/kg, IP	[39]
2017	IAPs	E3/ enzyme	PDE4	1137	**30**	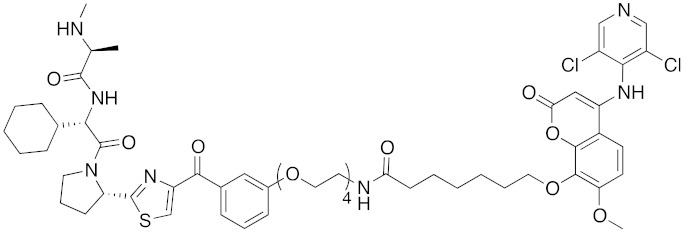	1 nM	-	[39]
2017	IAPs	E3/ others	BRD4	1056	**31**	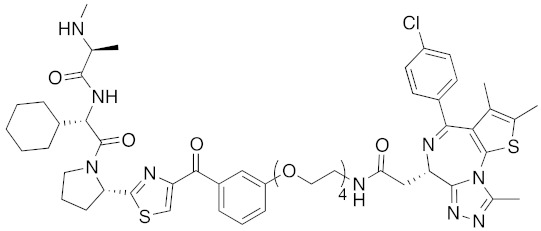	10 nM	-	[39]
2017	IAPs	E3/ enzyme	BCR-ABL	1070	**32**	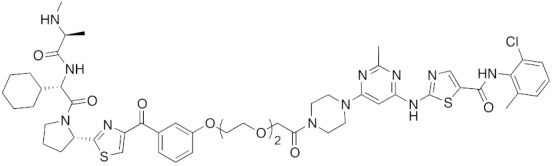	10 nM	-	[39]
2017	cIAP1	aggregation-prone (undruggable)	mHtt	720	**19**	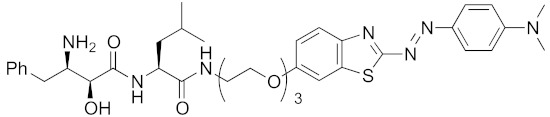	10 μM	-	[40]
2017	cIAP1	aggregation-prone (undruggable)	mHtt	662	**20**	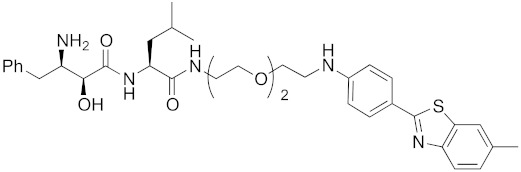	10 μM	-	[40]
2017	IAPs	enzyme (allosteric site)	BCR-ABL	1089	**35**	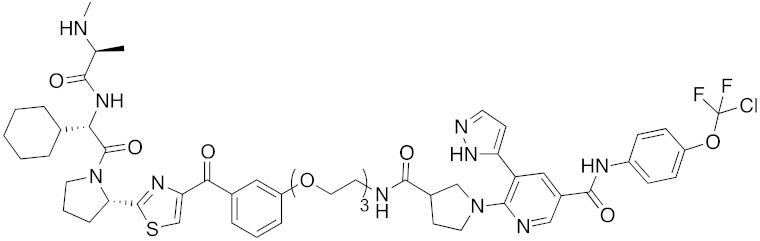	30 nM	-	[41]
2018	IAPs	aggregation-prone (undruggable)	mHtt	974	**27**	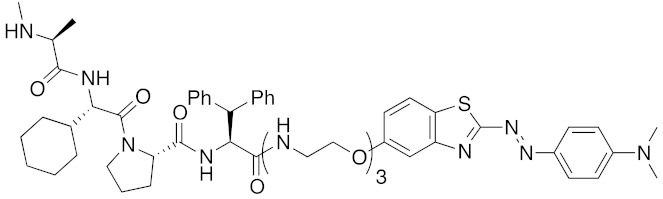	10 μM	-	[42]
2018	IAPs	E3/ receptor	ER	1122	**34**	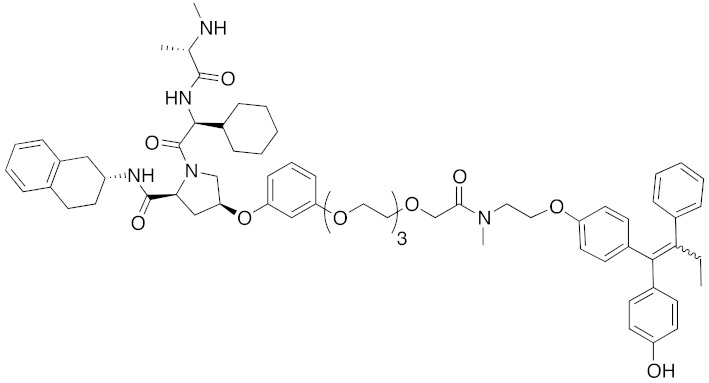	10 nM	30 mg/kg, ip	[43]
2018	IAPs	Tag	HisTag- fused protein	2116	**26**	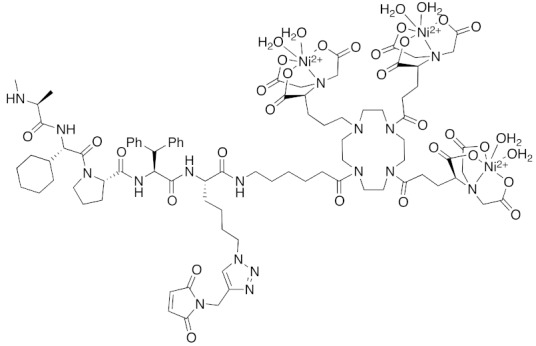	3 μM	-	[44]
2018	cIAP1	receptor	AR	864	**16**	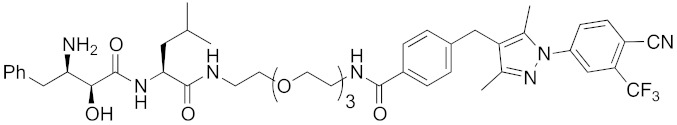	10 μM	-	[45]
2018	IAPs	receptor	AR	984	**33**	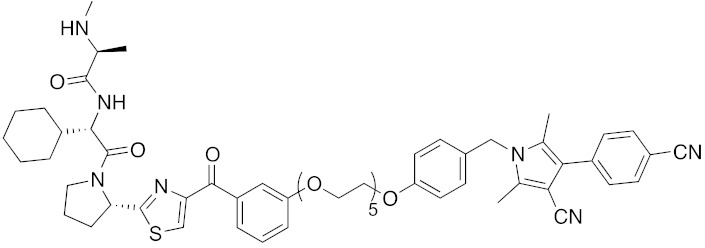	3 μM	-	[45]
2020	cIAP1	aggregation-prone (undruggable)	ataxin-3 ataxin-7 atrophin-1		**19, 20**	(The chemical structures are described above.)	10 μM	-	[46]

**Table 2:** Biological activities of bestatin analogs [56].

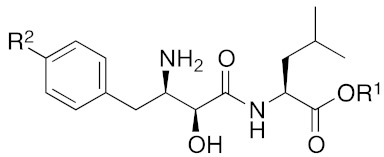					
**Compound**	**R^1^**	**R^2^**	**Aminopeptidase Inhibition pIC_50_**		**cIAP1 Degradation**
			**Ala**	**Arg**	
**4**	**H**	**H**	7.0	7.5	++
**5**	Me	H	6.9	5.7	+++
**6**	H	OH	7.1	8.7	+

The number of ‘+’ represents the strength of the activity.
